# Effectiveness, implementation, and monitoring variables of intermittent hypoxic bicycle training in patients recovered from COVID-19: The AEROBICOVID study

**DOI:** 10.3389/fphys.2022.977519

**Published:** 2022-11-02

**Authors:** Gabriel Peinado Costa, Alba Camacho-Cardenosa, Javier Brazo-Sayavera, Marcela Coffacci De Lima Viliod, Marta Camacho-Cardenosa, Yan Figueiredo Foresti, Carlos Dellavechia de Carvalho, Eugenio Merellano-Navarro, Marcelo Papoti, Átila Alexandre Trapé

**Affiliations:** ^1^ School of Physical Education and Sport of Ribeirao Preto, University of Sao Paulo, Ribeirao Preto, Brazil; ^2^ Department of Physical Education and Sport, Faculty of Sport Sciences, University of Granada, Granada, Spain; ^3^ Department of Sports and Computer Science, Universidad Pablo de Olavide, Seville, Spain; ^4^ Polo de Desarrollo Universitario EFISAL, Centro Universitario Regional Noreste, Universidad de la República, Rivera, Uruguay; ^5^ Clinical Management Unit of Endocrinology and Nutrition - GC17, Maimónides Biomedical Research Institute of Cordoba (IMIBIC), Reina Sofía University Hospital, Córdoba, Spain; ^6^ Ribeirao Preto Medical School, University of Sao Paulo, Ribeirao Preto, Brazil; ^7^ Departamento de Ciencias de la Actividad Física, Facultad de Ciencias de la Educación, Universidad Católica del Maule, Talca, Chile

**Keywords:** coronavirus infections, exercise, oxygen, physiologic monitoring, altitude, convalescence

## Abstract

Hypoxic exposure is safely associated with exercise for many pathological conditions, providing additional effects on health outcomes. COVID-19 is a new disease, so the physiological repercussions caused by exercise in affected patients and the safety of exposure to hypoxia in these conditions are still unknown. Due to the effects of the disease on the respiratory system and following the sequence of AEROBICOVID research work, this study aimed to evaluate the effectiveness, tolerance and acute safety of 24 bicycle training sessions performed under intermittent hypoxic conditions through analysis of peripheral oxyhemoglobin saturation (SpO_2_), heart rate (HR), rate of perceived exertion (RPE), blood lactate concentration ([La^−^]) and symptoms of acute mountain sickness in patients recovered from COVID-19. Participants were allocated to three training groups: the normoxia group (G_N_) remained in normoxia (inspired fraction of O_2_ (FiO_2_) of ∼20.9%, a city with 526 m altitude) for the entire session; the recovery hypoxia group (G_HR_) was exposed to hypoxia (FiO_2_ ∼13.5%, corresponding to 3,000 m altitude) all the time except during the effort; the hypoxia group (G_H_) trained in hypoxia (FiO_2_ ∼13.5%) throughout the session. The altitude simulation effectively reduced SpO_2_ mean with significant differences between groups G_N_, G_HR_, and G_H_, being 96.9(1.6), 95.1(3.1), and 87.7(6.5), respectively. Additionally, the proposed exercise and hypoxic stimulus was well-tolerated, since 93% of participants showed no or moderate acute mountain sickness symptoms; maintained nearly 80% of sets at target heart rate; and most frequently reporting session intensity as an RPE of “3” (moderate). The internal load calculation, analyzed through training impulse (TRIMP), calculated using HR [TRIMP_HR_ = HR * training volume (min)] and RPE [TRIMP_RPE_ = RPE * training volume (min)], showed no significant difference between groups. The current strategy effectively promoted the altitude simulation and monitoring variables, being well-tolerated and safely acute exposure, as the low Lake Louise scores and the stable HR, SpO_2_, and RPE values showed during the sessions.

## 1 Introduction

COVID-19, caused by the SARS-CoV-2 coronavirus, was initially characterized as an acute respiratory syndrome. However, after 526 million confirmed cases worldwide until June 2022 (World Health Organization, 2022), it is now known that not only during infection but also afterward, harmful effects can occur in the respiratory tract and lungs, as well as in the cardiovascular, nervous, and other systems (Gupta et al., 2020; Higgins et al., 2021).

Physical training, performed three to 5 days a week, lasting 15–45 min, continuously or intermittently, at moderate intensity, with heart rate and rate of perceived exertion to prescribe and monitor exercise, has been highly recommended to recover cardiopulmonary function (Bhatia et al., 2020; Laddu et al., 2020; Simpson and Katsanis, 2020) and improve the immune and nervous system, in patients affected by COVID-19 (Zhao et al., 2020; Calabrese et al., 2021). The adequate load results in beneficial adaptations, for example, a 6-week rehabilitation program that consisted of walking or treadmill exercise successfully improved respiratory capacity and endurance (Millet et al., 2016a). In contrast, people hospitalized due to COVID-19, who did not undergo exercise or rehabilitation when subjected to cardiopulmonary exercise test after 3 months of hospital discharge, still had a lower peak oxygen uptake (VO_2PEAK_) than expected (Skjørten et al., 2021).

It is essential to highlight the need to monitor and adequately control the training load. Proper exercise monitoring ensures that the patient/athlete adapts to the exercise program and minimizes the probability of developing an injury/illness or reaching an overtraining state (Halson, 2014). Training load refers to quantification by training volume and intensity. External load uses external intensity parameters, either resistance, speed, or power. In contrast, internal load quantifies physiological stress, through biomarkers, e.g., heart rate (HR), rate of perceived exertion (RPE), blood lactate concentration ([La^−^]), ventilation, or oxygen uptake (Halson, 2014).

Hypoxic training has been treated as a promising strategy for health (Camacho-Cardenosa et al., 2020; Behrendt et al., 2022), even with the difficulty of establishing an optimal hypoxia dose (Lizamore et al., 2016). Regarding hypoxia as an additional stimulus to exercise and the exercise workload, it is important to control the dose of exposure to hypoxia (Millet et al., 2016b; Soo et al., 2020; Behrendt et al., 2022). Several studies associated physical training and normobaric hypoxia, showing as moderate hypoxia (2,500 to 3,000 m simulated altitude) is a safe practice (Navarrete-Opazo and Mitchell, 2014), which presents significant results compared to normoxic situations, like fat mass reduction (Camacho-Cardenosa et al., 2018), lean mass increase (Matsumoto et al., 1999), cardiorespiratory improvement (Foster, 1998; Papoti et al., 2009; Camacho-Cardenosa et al., 2018). In addition, less mechanical stress with similar physiological changes have been shown in the special health condition population (Maggiorini et al., 1998; Pramsohler et al., 2017). A recent study by Carvalho et al. (2022) demonstrated that hypoxic exposure during the intervals between efforts can be an additional stimulus, represented by changes in HR and peripheral oxyhemoglobin saturation (SpO_2_) to training, without impairing the external load and the quality of the training sessions. This method allows maintenance of exercise quality and still has the benefits of exposure to intermittent hypoxia.

Therefore, hypoxic training could be considered a potential treatment to optimize recovery in COVID-19 convalescents. However, close monitoring is strongly recommended to ensure patient safety and enable its use in healthcare. Training monitoring using HR, SpO_2_, and RPE, among other variables, is widely used to identify desired and undesired responses (Maggiorini et al., 1998; Naeije, 2010; Chapman et al., 2011; Deb et al., 2018) while ensuring patient safety. Therefore, this study aimed to evaluate the effectiveness and acute safety of 24 training sessions of bicycle training associated with intermittent hypoxic through the description of SpO_2_, HR, RPE, [La^−^] analyses, in addition evaluating tolerance of hypoxic training in patients recovered from COVID-19.

## 2 Materials and methods

### 2.1 Design and participants

The present study follows the work of the AEROBICOVID project, a clinical trial controlled double-blind study were performed between September and December 2020, and details could be found elsewhere (Trapé et al., 2021). Participants aged 30–69 years and COVID-19 convalescents (with a positive diagnostic test) who had symptoms approximately 30 days since recovery from clinical signs or medical discharge were included. In addition, exclusion criteria were: individuals exposed to high altitude >1,500 m in the past 3 months, with significant physical limitations to perform the intervention, acute or chronic medical conditions without medical supervision, having anemia, using immunosuppressive drugs, being pregnant, hormone replacement, smokers, and excessive use of alcohol or drugs.

Participants were divided into three groups according to the combination of effort and recovery in normoxia and hypoxia conditions, i.e., training in normoxia and recovery in normoxia (G_N_); training in normoxia and recovery in hypoxia (G_HR_); and training in hypoxia and recovery in hypoxia (G_H_). The randomization was performed by four groups, with participants being directed to the control or one of three training groups. For the participants’ allocation to groups, it was taken into consideration the variables, gender, age, participant’s fitness level (result in the incremental test), and gravity during the disease (COVID-19). Blinding was done between the two research teams (evaluation and monitoring teams) and the participants.

The COVID-19 severity has been defined based on National Institutes of Health of United States of America criteria (Galloway et al., 2020; Gude et al., 2020). For, mild severity: have any symptoms of COVID-19, such as fever, cough, etc., but do not have shortness of breath or dyspnoea; moderate: have any symptoms of COVID-19 and have shortness of breath or dyspnoea; severe: have any symptoms of COVID-19 and need hospitalisation, but not intensive care; or critical: have any symptoms of COVID-19 and need hospitalisation and intensive care.

This study was approved by the Research Ethics Committees from the School of Physical Education and Sport of Ribeirao Preto—University of Sao Paulo (USP) and School of Pharmaceutical Sciences of Ribeirao Preto—USP (CAAE: 33783620.6.0000.5659; CAAE: 33783620.6.3001.5403, respectively) and registered in the Brazilian Registry of Clinical Trials (RBR-5d7hkv).

### 2.2 Instruments

The research experimental setup included two tents (Colorado Altitude Training Tent™, United States), with 12,000 L of air capacity, and a hypoxia generator (CAT-430™, Altitude Control Technologies, United States) for each tent (Trapé et al., 2021). There were participants from all three training groups around the tents and individual hoses directed towards the tent, all being covered by a tarp. Both tents had a tarp around them for blinding, hiding where the hoses (IVPU, vacuum air PU 1.1/2-cm) were attached to the tents. The hoses were located at the lower corners of the tents in all groups. In the GN, participants breathed ambient air because the hoses were on the side of the tents (outside) but covered by the tarp; in the GHR and GH, the participants breathed air with lower oxygen concentration, so the hoses were inside the tents, also covered by the tarp. The bicycles were positioned at a distance, which prevented visualization of the positioning of the hoses. At the end of the intervention, the participants received a questionnaire to answer between two options, whether they believed they belonged to the group in hypoxia or normoxia.

There were three types of bicycles, aiming to meet each participant’s limitations and individualities (e.g., joint pain, mobility difficulties, balance insecurity or uncomfortable seating): vertical spinning, vertical ergometric, and horizontal ergometric. In addition, each participant received a kit with a unidirectional mask (Air safety, Brazil) for individual use throughout intervention (Trapé et al., 2021) and a training diary. Each team member supervised up to three participants and was responsible for monitoring, collecting data, timing each training moment, and informing the participant what should be done.

### 2.3 Procedure

The hypoxia tents were initially designed for individual use, with the participant inside. The proposed new strategy, employed two tents and provided 16 participants simultaneously; four under hypoxia and four under normoxia.

Some procedures were carried out to avoid participants’ re-infection. Besides maintaining distance between them and each one receiving a unidirectional mask kit for individual use, all connections between the hose and masks remained submerged under hypochlorite for at least 30 minutes after use. These connection materials were washed with alcohol 70% and, after 30 minutes, were wrapped with a plastic paper.

Regarding the protection of the research team, each member received an individual face shield and used a disposable apron and cap beside the surgery mask. In addition, all disposable equipment was changed between sessions, and permanent equipment was sterilized with alcohol 70%.

A cardiopulmonary exercise incremental test was performed in a pendular cycle ergometer with mechanical braking (Ergometrica, Monark, Brazil) to determine the training intensity. Initially, the participants started a 5-min warm-up without any additional load; after that, the intensity was increased by 0.25 kp (∼15 W) every 2 minutes until the participant did not maintain the 60-rpm cadence or volitional exhaustion. Blood samples (25 μL) were collected from the earlobe at the end of each stage using previously calibrated heparinized capillaries. Blood samples were immediately dispensed and homogenized in microtubes containing 1% sodium fluoride for [La^−^] analysis using the YSI 2300 STAT analyzer (Yellow Springs, OH, United States). Concomitantly, HR and RPE were monitored at the end of each stage.

Anaerobic threshold 2 (AT2) was calculated for each subject from a blood lactate plot *versus* exercise intensity. Initially, the inflection point in the blood [La^−^] was determined by visual inspection. Subsequently, two linear regressions were plotted (before and after the inflection point), and the intercept of the lines $\left(y^{\prime }=y\right)=\left(\frac{\left(b^{\prime }-b\right)}{\left(a^{\prime }-a\right)}\right)$ was defined as AT2 (Matsumoto et al., 1999; Papoti et al., 2009). Having calculated AT2 intensity, the HR value referring to that intensity was assumed as 100%, and each participant should maintain the HR ranges during the workouts were calculated.

### 2.4 Intervention protocol

**FIGURE 1:**
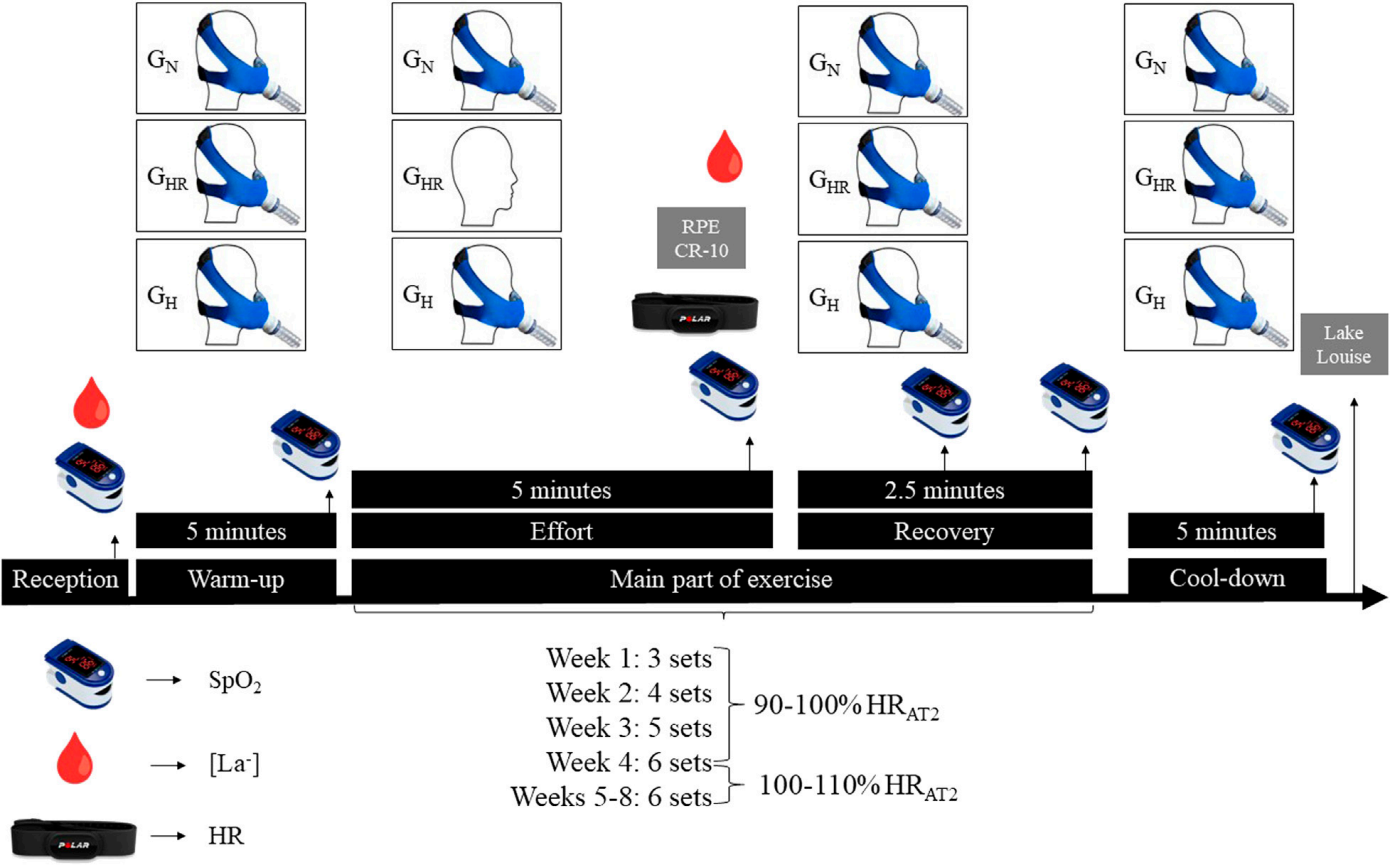
Experimental design of the training session. G_N_ = group in normoxia; G_HR_ = group in hypoxia recovery; G_H_ = group in hypoxia; RPE = rate of perceived exertion; SpO_2_ = peripheral oxyhemoglobin saturation; [La^−^] = blood lactate concentration; HR = heart rate; HR_AT2_ = heart rate relative to the anaerobic threshold 2.

The bicycle training was performed three times per week and consisted of three parts (warm-up, main part of exercise, and cool-down). The 5-min warm-up and the 3-min return to rest were performed in RPE 2, considered “easy” in a 0 to 10 scale (Foster et al., 2001). The main part of exercise was composed of three to six sets according to the established periods: each set was composed of 5-min efforts at an HR corresponding to 90%–100% (first to the fourth week) and 100%–110% (fifth to the eighth week) of the AT2, followed by a pause of 2.5 min to recover between sets (Figure 1).

The G_N_ and G_H_ used the mask during the entire workout (warm-up, effort, recovery and cool-down); G_HR_ used the mask all the time (warm-up, recovery and cool-down) except during efforts in the main part of exercise. At the hypoxic moments, participants were exposed to an inspired fraction of O_2_ (FiO_2_) of ∼13.5% (corresponding to 3,000 m altitude), being monitored inside the tent by an O_2_ sensor (Oxygen Sensor R-17MED, Teledyne Analytical Instruments, United States). At normoxic moments, participants breathed ambient air, with a FiO_2_ of ∼20.9% (a city with 526 m altitude).

### 2.5 Blood collection for lactate analysis

Blood collections for quantifying [La^−^] were performed at rest and at the end of each main part of exercise effort at weeks 2, 4, 6, and 8. [La^−^] was collected and determined according to the procedure previously described.

### 2.6 Exercise monitoring

The SpO_2_ was monitored by using a pulse oximeter (Portable, G-Tech Solutions, India) at rest (Rest), end of warm-up (W-Up), end of each effort (E-Effort), lowest value during recovery, end of each recovery (E-Recovery), and end of the cool-down (C-Down). For G_N_ and G_H_, the value during recovery was the mean value between the end of the effort and the recovery (M-R); for G_HR_, the value during recovery was the mean value among the end of the effort, the lowest value during recovery, and the end of recovery (M-R). The value during recovery was calculated individually for all training groups for each set and participant. The SpO_2_ value collected at the end of the last effort was used to calculate the average between this point and the end of recovery (M-CD).

The training diary contained the HR interval information to be used during training effort and spaces to annotate HR and RPE at the end of each set. HR was tracked in real-time and individually throughout the training. The SpO_2_ was recorded by the work team, positioning the device only when it was close to the moment of collection.

The monitoring of training intensity and the collection of values at the end of each effort of the main part of exercise were carried out by HR and RPE, using the Polar H10 tape and the scale adapted by Foster (1998), respectively.

### 2.7 Acute mountain sickness

The Lake Louise Scale (Maggiorini et al., 1998; Roach et al., 2018) was used to collect information related to acute mountain sickness and monitor acute responses to hypoxic exposure (headache, nausea/vomiting, fatigue, dizziness/light-headedness, and difficulty sleeping). The participants in the three training groups were asked to answer this questionnaire once a week. This questionnaire data should be used for descriptive purposes only and not to diagnose AMS, since there is a limitation because it is commonly applied in exposures longer than 6 hours.

### 2.8 Training zones

Using the HR data, the set percentage that participants remained below HR_AT2_ (<90%), within HR_AT2_ (90–100%; 100–110%), and above HR_AT2_ (>110%) were calculated. For RPE, the set percentage that participants remained below RPE_AT2_ (<RPE_AT2_), equal to RPE_AT2_ (=RPE_AT2_), and above RPE_AT2_ (>RPE_AT2_) were quantified. Regarding [La^−^], differently from AT2 calculations, the set percentage was evaluated in each training zone based upon fixed [La^−^], aiming to reduce potential variations arising from nutritional status. For each zone, it was defined that below 2 mmol refers to zone 1 (Z1); between 2 and 4 mmol, zone 2 (Z2); and above 4 mmol, zone 3 (Z3).

### 2.9 Training load

The internal training load was calculated by training impulse (TRIMP) in arbitrary units (a.u.) using the average of the HR [TRIMP_HR_ = HR * training volume (min)] and the RPE [TRIMP_RPE_ = RPE * training volume (min)] (Foster, 1998) from the main part of exercise.

### 2.10 Statistical analysis

The continuous and ordinal variables were expressed in basic descriptive statistics, mean (standard deviation) and median (minimum-maximum values). The association between categorical variables has been analyzed by chi-square. The Shapiro-Wilk and the Levene test were used to determine data normality and homogeneity, respectively. A two-way ANOVA with Tukey’s Post Hoc was performed to analyze and compare the groups and time variances. The significance level was set at 5% (*p* < 0.05) in all analyses, and the program used was JAMOVI version 2.3.

## 3 Results

### 3.1 Safety and effectiveness in the protocol implementation

**FIGURE 2:**
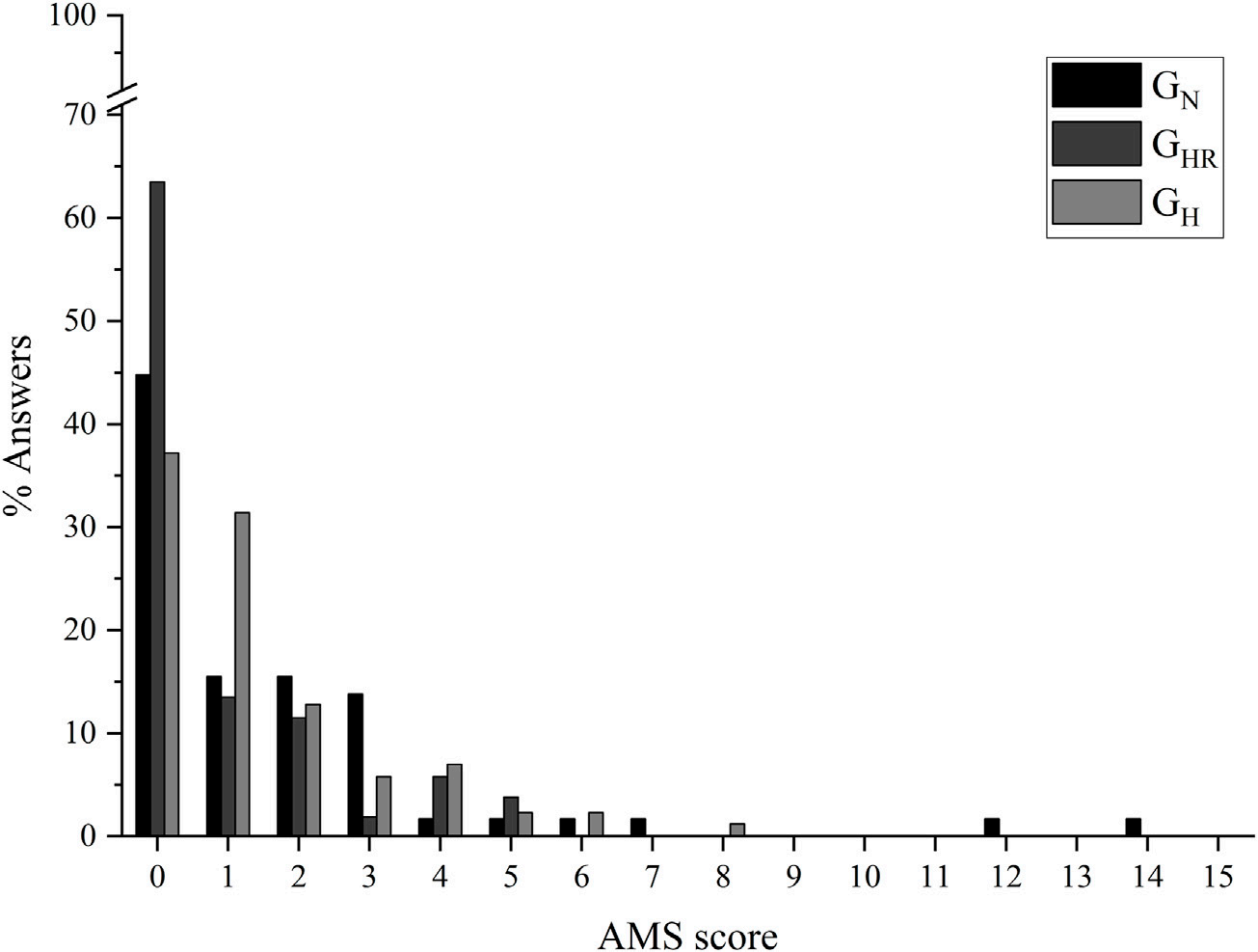
Acute mountain sickness symptom scores. AMS = acute mountain sickness; G_N_ = group in normoxia; G_HR_ = group in hypoxia recovery; G_H_ = group in hypoxia.

According to COVID-19 severity, the distribution of participants among the groups was 4 (G_N_), 4 (G_HR_), and 3 (G_H_) for mild severity; 11 (G_N_), 12 (G_HR_), and 17 (G_H_) for moderate; 3 (G_N_), 0 (G_HR_), and 1 (G_H_) for severe; and 3 (G_N_), 2 (G_HR_), and 1 (G_H_) for critical. Blinding appears was successful since 52.2% of participants incorrectly answered the perception of their group belonging. Furthermore, no association was observed between the hypoxic exposure models adopted and self-report symptoms of acute mountain sickness (*p* = 0.082). More than 93% of the participants showed no (score up to 2) or mild (score from 3 to 5) acute mountain sickness throughout Lake Louise Scale (Figure 2).

Table 1 shows peripheral oxyhemoglobin saturation (SpO_2_), heart rate (HR), and lactate concentration ([La^−^]) training responses to 8 weeks of the intervention. G_HR_ showed significantly lower values of SpO_2_ (*p* < 0.001) and percentage of maximum HR (%HR_MAX_) (*p* < 0.001) compared with G_N_. G_H_ showed significantly lower values of SpO_2_ and percentage of reserve HR (%HR_RES_) compared with G_N_ (SpO_2_, *p* < 0.001; %HR_RES_, *p* < 0.001) and compared with G_HR_ (SpO_2_, *p* < 0.001; %HR_RES_, *p* < 0.001), and showed significantly higher values of %HR_AT2_ compared with G_N_ (*p* < 0.001) and G_HR_ (*p* < 0.001). Still, %HR_MAX_ was significant higher in G_H_ compared with G_HR_ (*p* < 0.001).

**TABLE 1:** Training characterization of the main part of exercise through peripheral oxyhemoglobin saturation (SpO_2_), heart rate (HR), and the blood lactate concentration ([La^−^]) over the 8 weeks of intervention.

Group	n	SpO_2_	%HR_MAX_	%HR_AT2_	%HR_RES_	[La^−^] (mM)
G_N_	21	96.9 (1.6)	88.3 (8.0)	98.2 (8.3)	175.5 (46.3)	4.9 (1.8)
G_HR_	18	95.1 (3.1)*	87.0 (8.0)*	98.3 (8.5)	176.4 (41.8)	5.0 (2.1)
G_H_	22	87.7 (6.5)*#	88.6 (7.8)#	100.1 (7.8)*#	162.0 (27.0)*#	4.8 (2.0)

G_N_, group in normoxia; G_HR_, group in hypoxia recovery; G_H_, group in hypoxia; %HR_MAX_, relative maximum heart rate; %HR_AT2_ = relative heart rate to the anaerobic threshold 2; %HR_RES_, relative reserve heart rate; ^*^ = *p* < 0.05 compared to G_N_; ^#^ = *p* < 0.05 compared to G_HR_.

### 3.2 Monitoring variables

**FIGURE 3:**
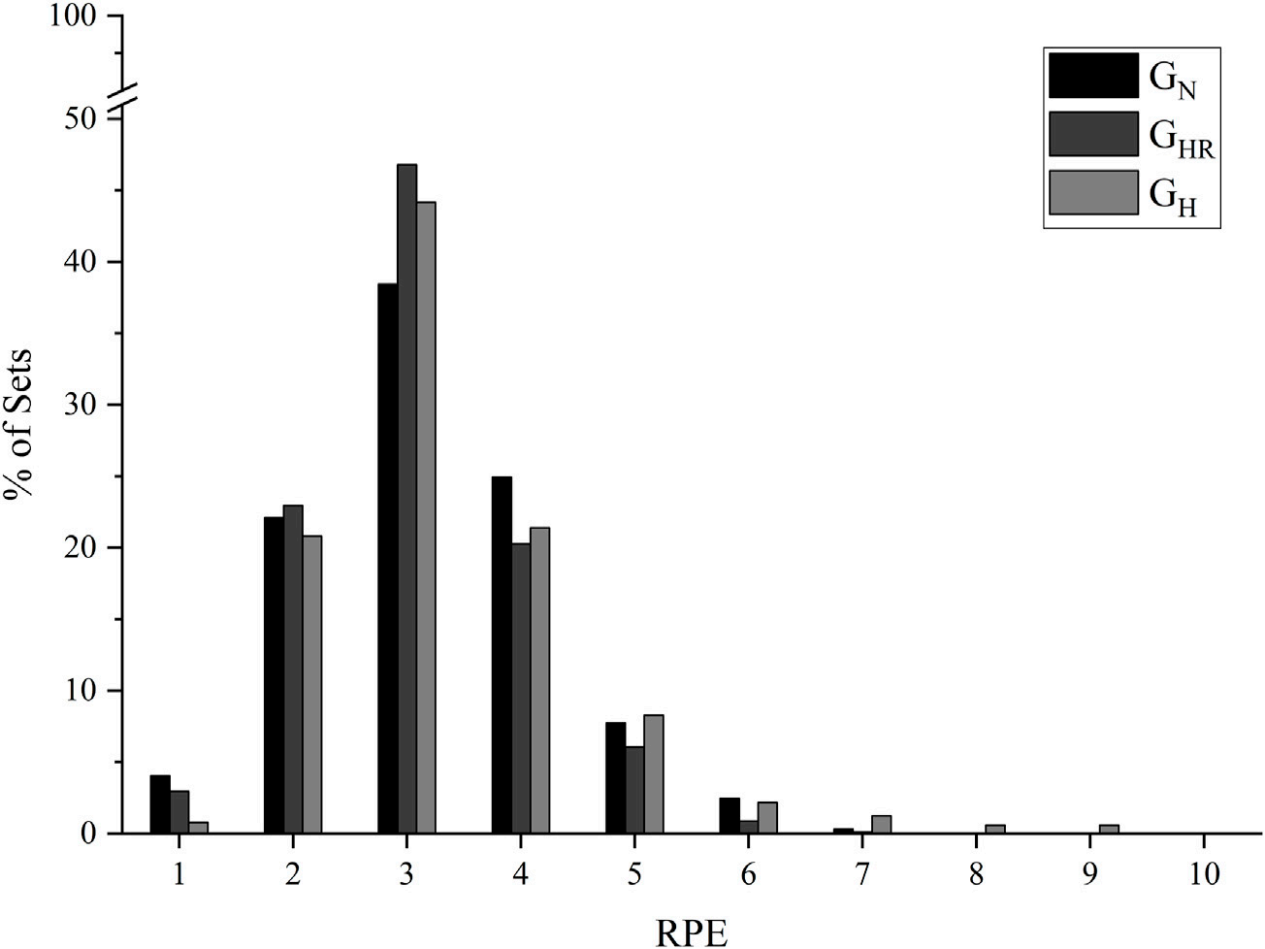
Frequency and distribution of the rate of perceived exertion (RPE) in the main part of exercise from the 8 weeks of intervention. G_N_ = group in normoxia; G_HR_ = group in hypoxia recovery; G_H_ = group in hypoxia.

In all groups, the most frequent RPE was “3” (moderate), and all groups maintained less than 10% above RPE “5" (hard) (Figure 3).

**FIGURE 4:**
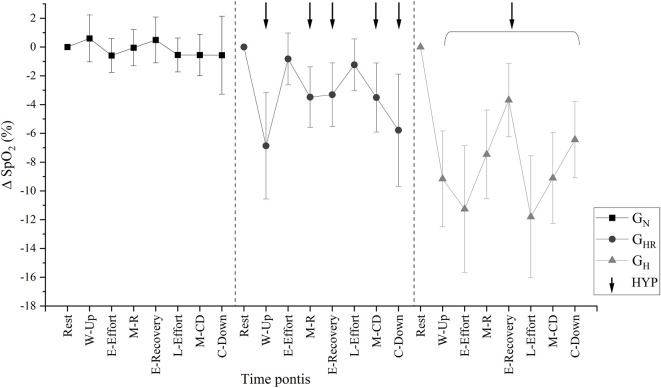
Average peripheral oxyhemoglobin saturation (SpO_2_) by delta kinetics of each set measurement from the first week of training. G_N_ = group in normoxia; G_HR_ = group in hypoxia recovery; G_H_ = group in hypoxia; HYP = times in hypoxia. Rest = rest; W-Up = warm-up; E-Effort = end effort; M-R = mean value during recovery; E-Recovery = end recovery; L-Effort = last effort; M-CD = average between “end of last effort” and “cool-down”; C-Down = cool-down.

The mean FiO_2_ inside the tent was 13.42 (0.34) %, while ambient air was stable at 20.9 (0.0) %. Figure 4 shows the kinetics by the mean delta SpO_2_ of each set measurement from the first week of training (rest, warm-up, end effort, during recovery, end recovery, last effort, during the return to cool-down, and after cool-down). SpO_2_ decreases according to hypoxia exposure in G_HR_, and a greater SpO_2_ reduction magnitude when the hypoxia is associated with the effort in G_H_.

Table 2 presents the intra and between groups analysis of the SpO_2_ during the first week of training. Regarding the analysis among groups at each time point, the G_HR_ showed a difference (*p* < 0.001) compared to the G_N_ according to hypoxic exposure in the warm-up, during recovery, end of recovery, and return to and after cool-down. Except at rest, G_H_ showed significantly lower (*p* < 0.001) SpO_2_ values compared with G_N_. In addition, SpO_2_ at the end of the effort, during recovery, during the last effort, and during cool-down were significantly lower (*p* < 0.005) in G_H_ compared with G_HR_. Regarding the intragroup analysis, all normoxic time points were significantly different from hypoxic time points (*p* < 0.001) in G_HR_. G_H_ group showed statistically significant differences (*p* < 0.001) in each point compared with the rest (Table 2). G_N_ showed no difference for SpO_2_ at any time point.

**TABLE 2:** Average peripheral oxyhemoglobin saturation (SpO_2_) of each measurement of a set from the first week of training.

Group	Time points							
	Rest	W-up	E-Effort	M-R	E-Recovery	L-effort	M-CD	C-down
G_N_	96.5 (2.8)	97.4 (0.9)	96.2 (1.9)	96.8 (1.4)	97.3 (1.2)	96.2 (1.9)	96.4 (1.9)	96.6 (2.4)
G_HR_	96.9 (1.6)	88.6 (4.6)*a	95.8 (2.3)^b^	93.5 (3.0)^*ac^	93.5 (3.6)^*abc^	95.5 (2.3)^b^	93.3 (3.1)^*ac^	91.2 (5.3)^*acef^
G_H_	95.9 (3.5)	87.0 (5.8)*a	85.1 (5.9)^*#a^	88.8 (4.3)^*#ac^	92.4 (3.8)^*abcd^	84.7 (5.4)^*#ade^	87.2 (4.4)^*#acef^	89.8 (4.5)^*acefg^

G_N_, group in normoxia; G_HR_, group in hypoxia recovery; G_H_, group in hypoxia. Rest = rest; W-Up = at the end of warm-up; E-Effort = at the end of end effort; M-R = mean value during recovery; E-Recovery = at the end of end recovery; L-Effort = at the end of last effort; M-CD, mean value during cool-down; C-Down = at the end of cool-down. ^*^ = *p* < 0.05 compared to G_N_; ^#^ = *p* < 0.05 compared to G_HR_. a = *p* < 0.05 compared to Rest; b = *p* < 0.05 compared to W-Up; c = *p* < 0.05 compared to E-Effort; d = *p* < 0.05 compared to M-R; e = *p* < 0.05 compared to E-Recovery; f = *p* < 0.05 compared to L-Effort; g = *p* < 0.05 compared to M-CD.

**FIGURE 5:**
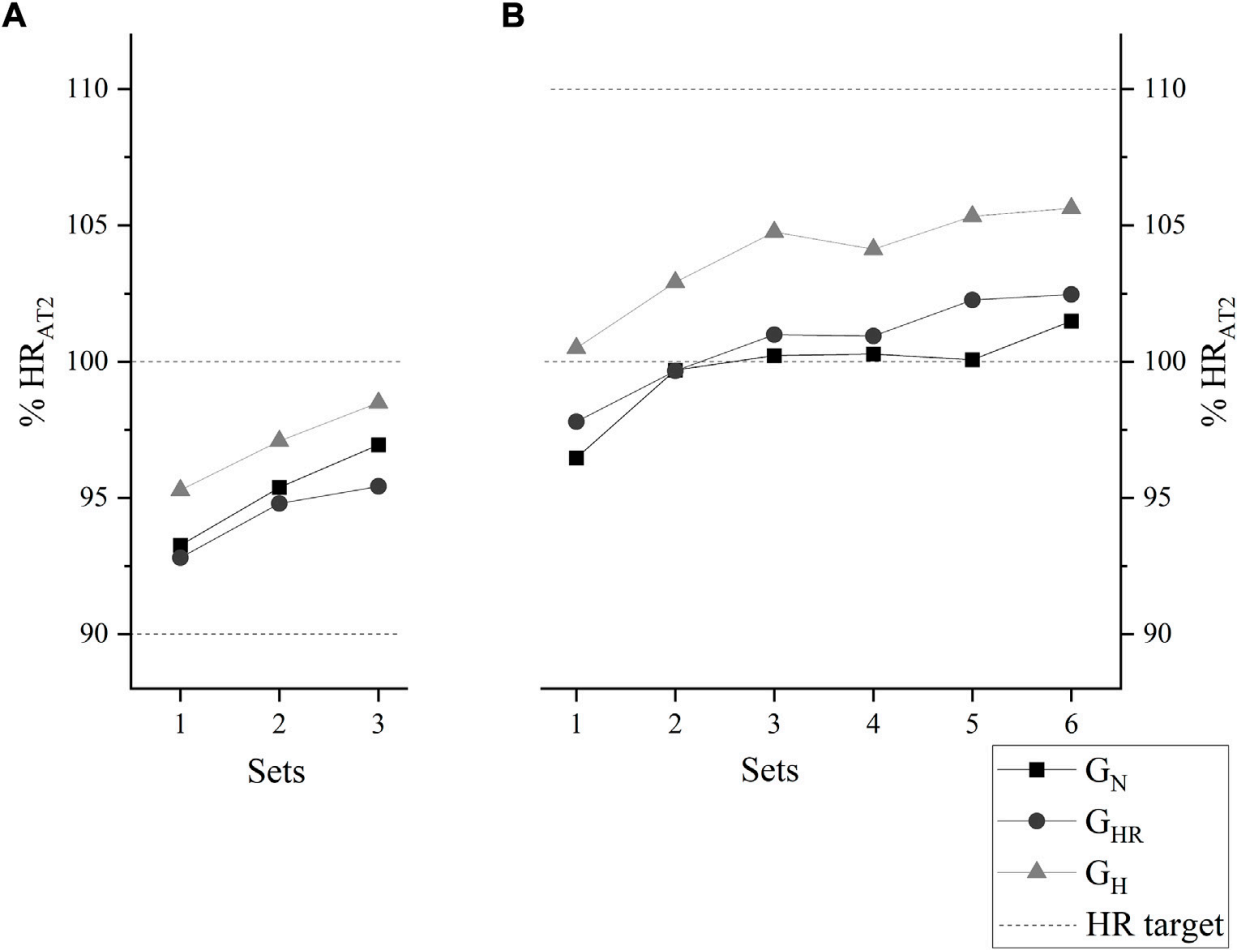
Average heart rate relative to the anaerobic threshold 2 (HR_AT2_) kinetics at the end of each set, at week 1, corresponding to 90%–100% **(A)**, and at week 8, corresponding to 100%–110% **(B)**. G_N_ = group in normoxia; G_HR_ = group in hypoxia recovery; G_H_ = group in hypoxia.

The participants predominantly stayed within the prescribed target (90%–100% of the AT2 in the first 4 weeks and 100%–110% from the fifth to the eighth week) during the sets performed (three sets on week 1 and six sets on week 8). No significant difference was observed among groups in HR values means over the sets neither at week 1 (Figure 5A) nor at week 8 (Figure 5B).

Analyzing the average HR of the 8 weeks of intervention, all groups remained predominantly in the HR_AT2_ range (90%–100% and 100%–110%). In addition, significant differences were found in the set percentage of these ranges compared with “below” (<90%) and “above” (>110%; *p* < 0.001). No differences were observed among groups (Table 3).

**TABLE 3:** Comparison of the set percentage below, within, and above the heart rate relative to the anaerobic threshold 2 (HR_AT2_).

Group	% Of sets below, within, and above the HR_AT2_			
	<90%	90%–100%	100%–110%	>110%
G_N_	16.6 (21.7)	41.8 (19.5)^a^	36.1 (25.9)^a^	5.5 (8.8)^bc^
G_HR_	14.7 (19.6)	46.7 (24.0)^a^	32.1 (21.9)	6.5 (13.2)^bc^
G_H_	9.5 (14.2)	43.4 (19.4)^a^	37.7 (18.9)^a^	9.5 (13.8)^bc^

G_N_, group in normoxia; G_HR_, group in hypoxia recovery; G_H_, group in hypoxia. <90% = below HR_AT2_; 90%–100% = within the proposed HR; >100% = above HR_AT2_. ^a^ = *p* < 0.05 compared to <90%; ^b^ = *p* < 0.05 compared to 90%–100%; ^c^ = *p* < 0.05 compared to 100%–110%.

Analyzing the average HR of the set percentage below, equal to, and above the RPE of the AT2 over the 8 weeks of intervention (Table 4), participants remained predominantly (*p* < 0.001) below the RPE_AT2_ and this percentage was significantly different compared with RPE related to the AT2 (*p* < 0.001) and RPE above AT2 (*p* < 0.001). No significant difference among the groups was observed.

**TABLE 4:** Comparison of the set percentage below, equal to, and above the heart rate relative to the anaerobic threshold 2 (HR_AT2_) over the 8 weeks of intervention.

Group	% Of sets below, equal to, and above RPE_AT2_		
	<RPE_AT2_	= REP_AT2_	>RPE_AT2_
G_N_	63.9 (36.1)	21.2 (19.5)^a^	14.1 (20.7)^a^
G_HR_	71.8 (28.1)	14.2 (14.3)^a^	13.6 (21.4)^a^
G_H_	81.6 (20.4)	12.2 (15.6)^a^	6.2 (11.7)^a^

G_N_, group in normoxia; G_HR_, group in hypoxia recovery; G_H_, group in hypoxia; <RPE_AT2_ = below RPE_AT2_; = RPE_AT2_ = equal to RPE_AT2_; >RPE_AT2_ = above RPE_AT2_. ^a^ = *p* < 0.05 compared to < RPE_AT2_.

Regarding absolute [La^−^], participants of all groups presented significantly higher [La^−^] in all sets compared with the rest (*p* < 0.001). However, no significant difference among groups was observed (Table 5).

**TABLE 5:** Mean absolute lactate concentration ([La^−^]) in each set over the 8 weeks of intervention.

Group	[La^−^] (mmol)						
	Rest	S1	S2	S3	S4	S5	S6
G_N_	1.4 (0.5)	3.7 (1.6)^a^	5.0 (1.9)^a^	5.2 (2.0)^a^	5.4 (2.1)^a^	5.0 (2.0)^a^	4.3 (1.9)^a^
G_HR_	1.5 (0.6)	4.1 (1.8)^a^	5.1 (2.3)^a^	5.1 (2.1)^a^	5.2 (2.1)^a^	5.2 (2.3)^a^	5.2 (2.3)^a^
G_H_	1.4 (0.5)	3.9 (1.3)^a^	5.0 (1.8)^a^	5.2 (1.6)^a^	5.3 (2.2)^a^	5.4 (1.9)^a^	5.1 (1.7)^a^

G_N_, group in normoxia; G_HR_, group in hypoxia recovery; G_H_, group in hypoxia; S = set. ^a^ = *p* < 0.05 compared to rest.

Table 6 shows the set percentage in each training zone according to [La^−^]. In all of them, the set percentage in Z3 was significantly higher (*p* < 0.001) compared with Z1. No significant difference among groups was observed.

**TABLE 6:** Set percentage in each training zone according to lactate concentration ([La^−^]) over the 8 weeks of intervention.

Group	Zones		
	%Z1	%Z2	%Z3
G_N_	4.2 (7.0)	36.7 (29.1)	59.1 (31.1)^a^
G_HR_	8.8 (26.4)	32.8 (38.4)	58.4 (41.0)^a^
G_H_	1.6 (4.8)	35.4 (35.5)	63.0 (37.6)^a^

G_N_, group in normoxia; G_HR_, group in hypoxia recovery; G_H_, group in hypoxia. %Z1 = % of sets below 2 mmol; %Z2 = % of sets between 2 and 4 mmol; %Z3 = % of sets above 4 mmol.

**FIGURE 6:**
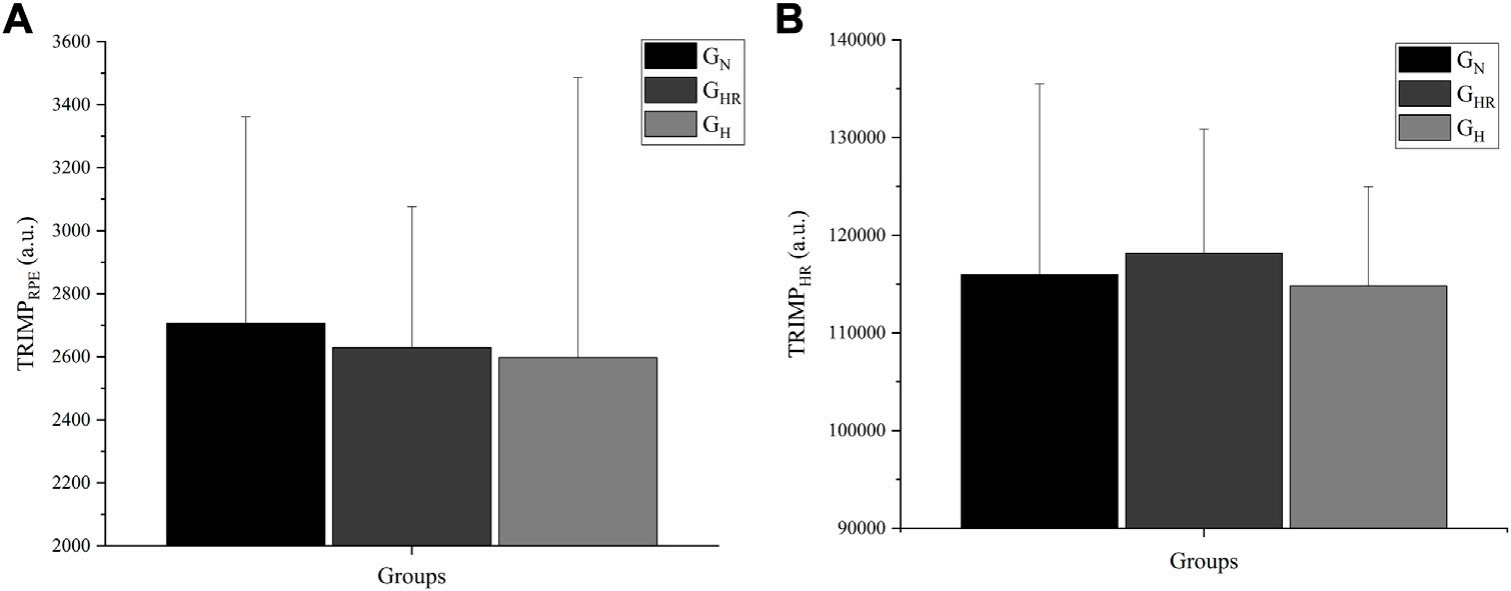
Mean TRIMP quantified by the rate of perceived exertion (RPE) **(A)** and heart rate (HR) **(B)** at the end of the 8-week intervention. G_N_ = group in normoxia; G_HR_ = group in hypoxia recovery; G_H_ = group in hypoxia; TRIMP_RPE_ = TRIMP quantified by RPE; TRIMP_HR_ = TRIMP quantified by HR.

Regarding the mean TRIMP quantified by RPE (Figure 6A) and HR (Figure 6B) at the end of the 8-week intervention, the groups did not present significant differences in the internal load of training.

## 4 Discussion

The present study aimed to describe acute responses of 24 bicycle training sessions combined with intermittent hypoxic through the SpO_2_, HR, RPE, [La^−^] analyses in patients recovered from COVID-19. Mean SpO_2_ was significantly different between groups, but no significant differences in HR_AT2_, RPE, [La^−^] means, and internal load calculated by TRIMP were shown.

### 4.1 Safety and effectiveness in the protocol implementation

The clinical trial was performed with three groups sharing a common training space. Although two groups were exposed to hypoxia, the blinding strategy—composed of tarps and similar individual mask systems—proved effective. It is important to emphasize that the error rate of the participants who incorrectly answered the perception of their group belonging was higher than 50%, even with 2/3 of participants under hypoxia.

Ensuring the safety of the intervention is essential to avoid harmful effects from altitude exposure exceeding 2,500 m, such as the increasingly frequent and intense occurrence of anorexia, nausea or vomiting, fatigue or weakness, dizziness or vertigo, or difficulty sleeping (Barry, 2003). A FiO_2_ corresponding to 3,000 m of altitude used in the present study proved to be well tolerated by both groups exposed to hypoxia (G_HR_ and G_H_): less than 4% self-reported moderate and 0% severe symptoms indicative of acute mountain sickness, measured by Lake Louise Scale (Roach et al., 2018). Furthermore, the most frequent score related to the acute mountain sickness, measured by Lake Louise Scale, was zero in the present study. It is important to highlight that this result is similar to the result presented by the group not exposed to hypoxia (G_N_), showing no or little hypoxic harmful effect.

The method to monitor the safety of hypoxic intervention is through the SpO_2_ (Bassovitch and Serebrovskaya, 2013), which must be below 80% (Richalet and Jean, 2017). The SpO_2_ average was significantly different between groups in the present study, showing the lowest values in the hypoxia-exposed groups. However, in both groups, the safety values of SpO_2_ remained. In a previous study, sedentary individuals exposed to 5,000 m simulated hypobaric hypoxia with and without combined bicycle exercise at 30% of VO_2MAX_ for 14-day showed a mean of SpO_2_ ranged 65.2 (9.9)% to 71.5 (7.3)% (Ricart et al., 2000). The lowest mean SpO_2_ in the present study was 84.7 (5.4)%, at the last effort of G_H_. Therefore, the system’s safety in hypoxic exposure might be verified based on this physiological variable.

Previous literature supports hypoxia in multiple health conditions (Millet et al., 2016a; Millet and Girard, 2017; Behrendt et al., 2022). More specifically, the safety and efficacy of physical exercises in recovering patients from severe acute respiratory syndrome (SARS) have already been identified but without a detailed description of monitoring variables (Lau et al., 2005). Lau et al. (2005) used HR to control that the exercise intensity in different ergometers ranged from 60% to 85% of HR_MAX_; participants from the three groups of this study maintained the HR mean at nearly 87.9% HR_MAX_. COVID-19 could carry limit patient functionality due to the generalized infection state and low mobility for a long time (Rooney et al., 2020). Moreover, even with natural fitness evolution through time, scores may remain lower than age-expected for as many as 2 years after recovery or hospital discharge (Rooney et al., 2020). Therefore, it becomes crucial to have a feasible intervention across all levels of physical function. Because of these aspects, the present research used three different bicycle models to attend to such a demand.

### 4.2 Monitoring variables

Other symptoms that may persist and limit patients in recovery, besides cardiorespiratory impairment, are the blood oxygen content and degree of dyspnea, which have been associated with higher death rates (Deng et al., 2020; Harapan et al., 2020). However, physical exercise must be performed while monitoring variables for safety and the intervention’s effectiveness.

For equalizing stimulus between participants, individually calculated internal parameters (HR, RPE, and [La^−^]) from the incremental test were used to control intensity. In the present study, the prescribed target HR was 90%–110% HR_AT2_, and participants maintained over 77% of the sets as stipulated. Therefore, results of the present study identify the possibility that individuals recovering from COVID-19 can tolerate a relatively high exercise intensity based on target HR or similar.

Among various hypoxia exposure methods (Bassovitch and Serebrovskaya, 2013), this study has used normobaric hypoxia. A concern with this system was the number of participants breathing in the same tent and the air available at the reservoir. Still, the effectiveness of the simulation system used is noted by the mean values of all interventions significantly different between groups and the significant difference between normoxia (G_N_: all times; G_HR_: effort; G_H_: none) and hypoxia (G_N_: none; G_HR_: warm-up, recovery, and cool-down; G_H_: all times) moments within the group. Although there were differences in SpO_2_ between groups, both hypoxic exposure models (G_HR_ and G_H_) did not result in severe discomfort. In addition, the most frequently reported RPE was “3” (moderate perceived exertion) in all groups, and 63.9%–81.6% of the sets were maintained below the RPE_AT2_, without significant differences between groups. In a study that instructed participants to keep a RPE between “hard” and “very hard” during exercise bicycle ergometer with an intensity of approximately 80% of maximum work rate, lower values were observed in time to exhaustion, VO_2_, and minute ventilation when performed under hypoxia (FiO_2_ = 11.4%) (Jeffries et al., 2019). This finding reinforces that external load decreases when hypoxic exposure occurs, and the internal load parameter is used to equalize groups.

Beyond the RPE, the [La^−^] without difference among sets and between groups demonstrated that intensity 90%–110% HR_AT2_ and effort/pause ratio are feasible since nearly 80% of sets were performed at HR target; and provide a relative physiological steady state. During hypoxia, aerobic energy contribution decreases, which may cause metabolic acidosis and performance reduction (Bowtell et al., 2014). This result could overload already weakened systems of COVID-19 recovery people and result in different responses between groups. However, the non-significant differences could indicate no acidosis, preventing physiological overload. Nam and Park (Nam and Park, 2020) found a significant increase in [La^−^] values (near 9 mmol) and blood pH decrease according to FiO_2_ reduction (20.9%–16.5% and 12.8%), comparing three exposure models associated with 30 min of continuous bicycle ergometer exercise at 80% intensity of HR_MAX_. The interaction between intensity and volume of exercise and FiO_2_ results in these reactions: lower FiO_2_ decreases VO_2_, leading to greater energy contribution from glycolytic pathway and subsequent higher hydrogen ion levels; and, at some point, acid-base balance does occur, and besides lactate not causing metabolic acidosis, it is a biomarker of this phenomenon (Lühker et al., 2017). In the present study, mean HR relative to maximal was between 87% and 88.6%, but with a delta of [La^−^] lower than 1 mmol, indicating a relative physiological steady state. Although both values of relative HR and overall effort volume (6 sets of 5 min) were similar, we employed intermittent efforts, with an effort/pause ratio of 2:1. The intermittent effort appears to be important to avoid metabolic acidosis.

Deb et al. (2018), in a systematic review with meta-regression, demonstrated that continuous or intermittent exercises, with efforts longer than 2 min, under simulated or environmental hypoxia (above 1,000 m) cause decreased performance and, consequently, lower external load. This reduced performance is also associated with the magnitude of desaturation, showing a VO_2MAX_ reduction of 2% for every SpO_2_ reduction of 1% (Chapman et al., 2011). To maintain an equal external load from normoxia to hypoxia, HR tends to increase because of increased pulmonary vascular resistance and maintenance of cardiac output (Naeije, 2010). Zoll et al. (2006) showed a significant reduction in external load compared to a normoxic effort during a second ventilatory threshold intensity effort (similar to the AT2) under hypoxia (FiO_2_ = 14.5%) at the same relative HR. Based on the magnitude of G_H_ desaturation, the exposure time to hypoxia, no significant difference in relative HR, and the internal load parameters (TRIMP_HR_ and TRIMP_RPE_), it can be presumed that there was a reduction in external load on G_H_. This reduction may negatively affect athletes, but it may benefit individuals with special health conditions because the ergogenic effects would be achieved with less mechanical stress (Girard et al., 2021). These results demonstrate that this kind of intervention is more suitable for physical limitation or rehabilitation (Maggiorini et al., 1998). Therefore, the G_HR_ arises with the proposal of performing the efforts in normoxia and recovery in hypoxia, obtaining the ergogenic effects of hypoxia exposure without reducing the external load.

Some limitations should be described. First, the load was only quantified through TRIMP_HR_ and TRIMP_RPE_, internal load quantification methods, and without external load quantification. This can be explained through the number and diversity of bicycle ergometers required for the study according to the participant’s limitations. The sample size could also be considered a limitation (described in the study protocol) but explained by the health epidemic scenario where the study was carried out and the complexity of the experimental design. Nevertheless, the study had extensive data collection to provide a robust understanding of physiological responses, having 15,826 SpO_2_ values, 6,036 HR values, and 7,198 RPE values.

It is important to highlight that the present study is original and provides detailed descriptions, never seen before in severe acute respiratory syndrome disease, of the materials, methods, and physiological responses expected by a protocol that proved safe and effective. These descriptions and results bring the literature closer to clinical and professional practice, enabling the protocol’s replication. Furthermore, once the effectiveness, tolerance and acute safety of 24 training sessions have been demonstrated, technologies can be developed to decrease the large costs required by such protocol and a portable device, enabling other exercises to explore other conditioning and coordinative capacities.

## 5 Conclusion

The current strategy effectively promoted altitude simulation. This strategy, it has been shown as well-tolerated and acutely safe exposure during the sessions, as indicated by the low acute mountain sickness and the stable HR and SpO_2_ values. Furthermore, it was possible to monitor exercise-induced physiological responses under three different environmental conditions in recovered patients from COVID-19 with persistent symptoms. For future research, an improved quantifying of the external load is suggested. The findings presented in this study may strengthen the tolerance and safety of developing hypoxia combined with physical exercise interventions for COVID-19 convalescents and developing technologies that facilitate accessibility.

## Data Availability

The original contributions presented in the study are included in the article/Supplementary Material, further inquiries can be directed to the corresponding author.
